# LAPAROSCOPICALLY ASSISTED ANORECTOPLASTY AND THE USE OF THE BIPOLAR DEVICE TO SEAL THE RECTAL URINARY FISTULA

**DOI:** 10.1590/0102-6720201600030016

**Published:** 2016

**Authors:** Robson Azevedo DUTRA, Adriana Cartafina Perez BOSCOLLO

**Affiliations:** Department of Pediatric Surgery, Federal University of Triangulo Mineiro, Uberaba, MG, Brazil

**Keywords:** Anorectal anomaly, Videosurgery, Bipolar sealing.

## Abstract

**Background:**

The anorectal anomalies consist in a complex group of birth defects. Laparoscopic-assisted anorectoplasty improved visualization of the rectal fistula and the ability to place the pull-through segment within the elevator muscle complex with minimal dissection. There is no consensus on how the fistula should be managed.

**Aim:**

To evaluate the laparoscopic-assisted anorectoplasty and the treatment of the rectal urinary fistula by a bipolar sealing device.

**Method:**

It was performed according to the original description by Georgeson^1^. Was used 10 mm infraumbilical access portal for 30º optics. The pneumoperitoneum was established with pressure 8-10 cm H_2_O. Two additional trocars of 5 mm were placed on the right and left of the umbilicus. The dissection started on peritoneal reflection using Ligasure(r). With the reduction in the diameter of the distal rectum was identified the fistula to the urinary tract. The location of the new anus was defined by the location of the external anal sphincter muscle complex, using electro muscle stimulator externally. Finally, it was made an anastomosis between the rectum and the new location of the anus. A Foley urethral probe was left for seven days.

**Results:**

Seven males were operated, six with rectoprostatic and one with rectovesical fistula. The follow-up period ranged from one to four years. The last two patients operated underwent bipolar sealing of the fistula between the rectum and urethra without sutures or surgical ligation. No evidence of urethral leaks was identified.

**Conclusion:**

There are benefits of the laparoscopic-assisted anorectoplasty for the treatment of anorectal anomaly. The use of a bipolar energy source that seals the rectal urinary fistula has provided a significant decrease in the operating time and made the procedure be more elegant.

## RESUMO

**Racional:**

As anomalias anorretais consistem de um grupo complexo de defeitos congênitos. A anorretoplastia laparoscópica permite melhor visualização da fístula retourinária e propicia o posicionamento do reto abaixado dentro do complexo muscular do elevador do ânus com mínima dissecção. Não há consenso na literatura sobre o melhor tratamento dessa fístula.

**Objetivo:**

Avaliar a anorretoplastia laparoscópica e o selamento bipolar da fístula retourinária.

**Método:**

Ela foi realizada de acordo com a descrição original de Georgeson^1^. Utilizou-se o acesso infraumbilical com portal de 10 mm para a ótica de 30º. O pneumoperitônio foi estabelecido com pressão de 8-10 cm de H_2_O. Dois trocárteres adicionais de 5 mm foram colocados à direita e à esquerda da cicatriz umbilical. A dissecção foi iniciada na reflexão peritoneal usando Ligasure^(r)^. Com a redução do calibre do reto distalmente, foi identificada a fístula para a o trato urinário. O local do novo ânus foi definido por meio da localização do complexo muscular do esfíncter anal externo, utilizando-se estimulador eletro muscular externamente. Por fim, foi confeccionada uma anastomose entre o reto e o novo local do ânus. Uma sonda uretral de Foley foi deixada durante sete dias.

**Resultados:**

Sete meninos foram operados, seis com fístula retoprostática e um retovesical. O período de seguimento variou de um a quatro anos. Os dois últimos pacientes operados foram submetidos ao selamento bipolar da fístula entre o reto e a uretra, sem suturas ou ligadura cirúrgica com pontos. No seguimento em longo prazo não houve evidências de fístulas urinárias.

**Conclusão:**

Há benefícios da anorretoplastia laparoscópica para o tratamento de anomalia anorretal. O uso de uma fonte de energia bipolar que promova o selamento da fístula retourinária propiciou redução significativa do tempo cirúrgico e tornou o procedimento mais elegante.

## INTRODUCTION

The anorectal anomalies consist of a complex group of birth defects with a broad variety of expressions. The incidence is of 1 in 5.000 births and it is due to an abnormalities of embryological development of the anus, rectum and/or urogenital tract with high morbidity and mortality rate^3^ ^,^ ^4^. The most common defect in male is rectourethral fistula and rectovestibular fistula in female. There are different techniques available for correction of the anomaly with their specific morbidity. The treatment is based on the topography of the abnormality and the central issue is the pull-through of the rectum and reconstruction of the anus and sphincter complex^8^. The posterior sagittal anorectoplasty (PSARP) has been the most commonly used technique for the repair of high and intermediate anorectal malformations since the early 1980s^3^. In 2000, Georgeson et al.^1^ introduced the laparoscopic-assisted anorectoplasty technique (LAARP) that has gained interest because of improved visualization of the rectal fistula and surrounding structures, proper placement of the rectum without division of the muscle complex and minimal abdominal and perineal wounds.The treatment of the rectourethral fistula is controversial. Clips, sutures ligation or simple division^4^ ^,^ ^7^ ^,^ ^9^ may be used. 

The aim of this study was to demonstrate the surgical treatment of high anorectal anomaly by laparoscopic surgery and to present the treatment of retourinary fistula through sealing process with bipolar device.

## METHODS

A retrospective analysis was based on medical records revision from March 2011 to January of 2016. Seven male patients aged from eight months to one year and six month at moment of the surgery were operated. Six had rectoprostatic and one rectovesical fistula. The follow-up period ranged from one to four years. The last two patients operated underwent bipolar sealing of the fistula between the rectum and urethra without sutures or surgical ligation with points.

### Surgical technique

**FIGURE 1:**
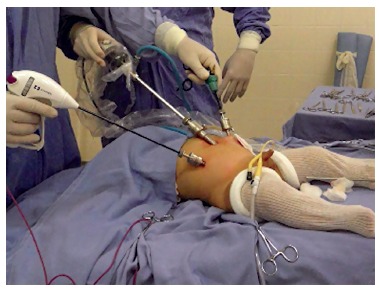
Child in operating room with one umbilicus trocar of 10mm and two of 5 mm on the right and left flanks

**FIGURE 2:**
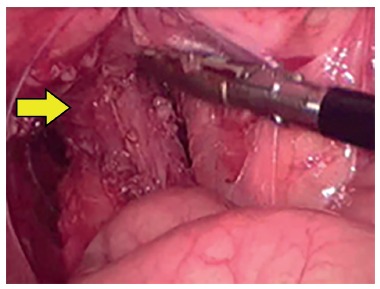
Rectal dissection into the prostatic urethra (arrow)

**FIGURE 3:**
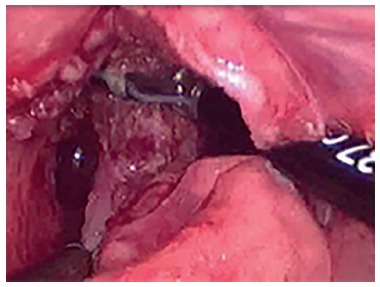
Sealing of rectal urinary fistula

**FIGURE 4:**
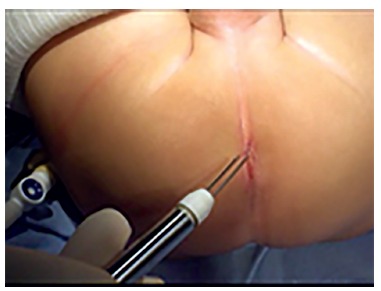
Largest contraction location point of the external sphincter using skin stimulator

**FIGURE 5:**
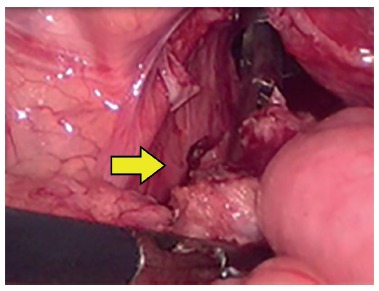
Laparoscopic forceps (arrow) introduced within a 10 mm trocar at the marked point orientated by the stimulator (new anus), lowering the rectum

**FIGURE 6:**
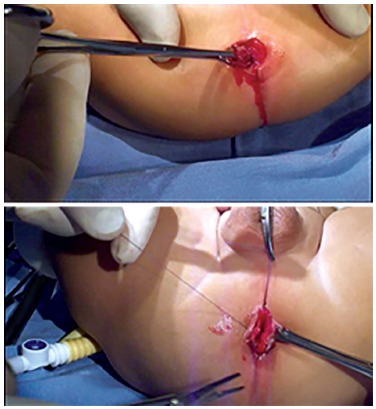
A) Rectum downloaded within the anorectal muscle complex; B) new anus

LAARP was carried out according to Georgeson's description^1^ with minor modifications. Was used the umbilicus as a camera port. Pneumoperitoneum was established with a pressure of 8-10 cm H_2_O. Two additional 5-mm trocars were placed at right and left side of the umbilicus (Figure 1). Laparoscopic rectal dissection was begun at the peritoneal reflection using the bipolar device (Ligasure^(r)^, Valleylab, Boulder, CO). The distal mesorectum was divided and the bipolar dissection continued anteriorly and laterally on the rectal wall. As the rectum was tapered distally, the fistula to the urethra was identified (Figure 2). In the first five cases, it was sutured with absorbable stitches and cut. In the last two cases, the fistula was merely sealed at the insertion on the posterior urethra (Figure 3). The location of the new anus was defined by the location of the external anal sphincter complex, that is the region with the best muscle contraction, using a transcutaneous electrostimulation (Penã Muscle Stimulator, Radionics Corporation, Burlington, MA) (Figure 4). A Veress needle was introduced at this point and its path identified near and parallel to the urinary tract using a laparoscopic view. Then, the muscular complex was dilated with 5 and 10 mm trocars and the rectum was pulled through via a 10-mm trocar (Figure 5). Finally, an anastomosis was made between the rectum and the new anus (Figure 6). A diversion of urine through a Foley catheter was left for seven days.

## RESULTS

Abdominal surgery was conducted exclusively via laparoscopic, with total surgical time (abdominal and perineal time) ranging from 90 (cases bipolar sealing) to 120 min (standard surgical ligation). Two patients had complications, one with a rectal perforation that was treated with two layers sutures and the other had a mucosal anal prolapse that was surgically corrected later. The last two patients had their fistulas sealed with the bipolar device. The long term follow up identified no complications. Five children older than three years were evaluated according to the Krickenbeck Consensus^2^. The criteria evaluated were the perception of bowel movements, the presence of soiling, and constipation. Four children had occasional soiling (good result) and one child frequent soiling (bad result). The last two children submitted to the sealing of the fistula were under three years old and were not evaluated. No evidence of urethral leaks was identified. 

## DISCUSSION

The benefits of LAARP for the treatment of anorectal anomaly includes a lack of necessity for complex sphincter division, better compliance of the external sphincter, excellent visualization of rectal fistula and the surrounding structures, preservation of the distal rectum with accurate placement of the rectum within the elevator anus and the external anal sphincter muscle complex. Indeed, laparoscopy promotes the improvement of rectal resting pressure and anorectal inhibitory reflex^9^. It is a less invasive procedure and the results are similar to the traditional surgery (PSARP) with better postoperative recovery and shorter hospital stay. In males, it is indicated for the treatment of rectoprostatic and rectovesical fistulas. In female the minimally invasive surgery is rarely indicated; the treatment of rectal urinary fistula is usually carried out in a conventional manner by surgical ligation. 

The sealing bipolar device system is used frequently to seal vessels. This device monitors the energy expended while denaturing the collagen and elastin within the vessels walls. During the cooling phase of the cycle, cross linking re-occurs creating a new seal. It was demonstrated in an experimental model the possibility of intestinal loop anastomosis using the sealing bipolar device, given the rich content of collagen in the intestinal submucosal wall, sealing the juxtaposed edges^5^. Thus, is postulated the sealing of the rectum urinary fistula and urethral catheterization for seven days. After more than one year of follow up, these children had no complications.

## CONCLUSION

There are benefits of LAARP for the treatment of anorectal anomaly. The use of a bipolar energy source that seals the rectal urinary fistula has provided a significant decrease in the operating time and turned the procedure more elegant.

## References

[1] Georgeson KE, Inge TH, Albanese CT (2000). Laparoscopically assisted anorectal pull-through for high imperforate anus-a new technique. J Pediatr Surg.

[2] Holschneider AM, Hudson JM (2006). Anorectal malformations in children.

[3] Pena A, De Vries PA (1982). Posterior sagittal anorectoplasty: important technical considerations and new applications. J Pediatr Surg.

[4] Rollins MD, Downey EC, Meyers RL, Scaife ER (2009). Division of the fistula in laparoscopic-assisted repair of anorectal malformations-are clips or ties necessary?. Journal of Pediatric Surgery.

[5] Smulders JF, de Hingh IHJT, Stavast J, Jackimowicz JJ (2007). Exploring new technologies to facilitate laparoscopic surgery: creating intestinal anastomoses without sutures or staples, using a radio-frequency-energy-driven bipolar fusion device. Surg Endosc.

[6] Sydorak RM, Albanese CT (2002). Laparoscopic repair of high imperforate anus. Semin Pediatr Surg.

[7] Van der Zee DC, Dik P, Beek FJ. (2013). Laparoscopy-assisted Anorectal Pull-through in Anorectal Malformations. A Reappraisal World J Surg.

[8] Vick LR, Gosche JR, Boulanger SC (2007). Primary laparoscopic repair of high imperforate anus in neonatal males. J Pediatr Surg.

[9] Yang J, Zhang W, Feng J (2009). Comparison of clinical outcomes and anorectal manometry in patients with congenital anorectal malformations treated with posterior sagittal anorectoplasty and laparoscopically assisted anorectal pull through. J Pediatr Surg.

